# The Effect of Inelastic Compression Wraps on the Quality of Life of People with Chronic Venous Insufficiency: A Single-Center, Single-Arm, Prospective Study

**DOI:** 10.3390/jcm14072275

**Published:** 2025-03-26

**Authors:** Abby Hargis Smits, Marita Yaghi, Narges Maskan Bermudez, Hadar Lev-Tov

**Affiliations:** 1 Macon and Joan Brock Virginia Health Sciences, Eastern Virginia Medical School, Old Dominion University, Norfolk, VA 23507, USA; 2 Dr. Phillip Frost Department of Dermatology and Cutaneous Surgery, University of Miami Leonard M. Miller School of Medicine, Miami, FL 33136, USA

**Keywords:** chronic venous insufficiency, compression stockings, compression wrap, venous dermatitis, lipodermatosclerosis, atrophie blanche

## Abstract

**Background/Objectives**: First line therapy for all manifestations of chronic venous insufficiency (CVI) is compression. However, patients frequently report dissatisfaction with compression stockings. Therefore, there is a need to find alternative therapeutic options that can promote compliance. Here, we investigate the impact of the novel, inelastic compression wrap device on quality of life (QoL) in patients with CVI who have failed therapy with compression stockings in the past. **Methods**: We conducted a six-week, open-label, single-center, non-blinded, prospective cohort study. The primary endpoint was the change in QoL over 6 weeks as measured by the Chronic Venous Disease Quality of Life Questionnaire (CIVIQ-20). **Results**: Thirty patients completed the study. Twenty-five (83.3%) reported wearing the compression device most of the time. At the six-week follow-up, CIVIQ-20 scores improved on average 12.123 ± 21.06 points on a 100-point scale (*p* = 0.0019). Calf circumference decreased on average 1.3 cm ± 2.21 cm (*p* = 0.0009). Measured on a ten-point scale, average itch decreased 1.9 ± 2.63 points (*p* = 0.0008) and reported levels of the worst itch decreased on average 2.73 ± 3.63 points (*p* = 0.0001). The Venous Clinical Severity Scoring scores decreased on average by 1.276 ± 2.297 points (*p* = 0.0029). **Conclusions**: Compression stockings remain the mainstay of treatment for advanced cutaneous manifestations of CVI. However, we demonstrated that the novel inelastic compression device offers an alternative and may improve QoL, compliance, and clinical venous symptoms in a safe manner in people who could not tolerate compression stockings.

## 1. Introduction

Chronic venous disease (CVD) is a consequence of functional abnormalities of the venous system [1]. Several risk factors for CVD have been identified including genetics, a family history of varicose veins, the female sex, advancing age, and several lifestyle risk factors that also play a role, such as a history of limited physical activity, periods of prolonged standing, obesity, a history of pregnancy, and smoking [2,3,4,5,6]. Advanced CVD is termed chronic venous insufficiency (CVI), which is the clinical reflection of chronic venous hypertension in the lower extremities [7]. CVI is often associated with the symptoms of pain and itch. The stages of CVI are most recognizable visually as patterns on the skin and reflect disease severity and duration. The cutaneous signs of CVI include lower extremity edema, hyperpigmentation, eczematous dermatitis, corona phlebectatica, lipodermatosclerosis, atrophie blanche, and ulceration. The first line therapy for all manifestations of CVD and CVI is compression, most often with compression stockings of 20–30 mm Hg pressure (30–40 mm Hg for venous ulceration) [8,9]. Compression therapy is conservative and appropriate for most patients regardless of factors such as age and comorbid conditions. However, patients frequently report dissatisfaction with compression stockings for a variety of reasons [10]. Some of the reasons why patients have reported non-compliance with compression stockings include that they were ineffective, painful or “cut off circulation”, too hot to wear, poor cosmetic appearance, cause limb soreness, and are difficult to put on without help, among others [10]. It has been reported that up to 63% of patients with CVD who have been prescribed compression stockings no longer use them [10]. Patients who are non-complaint with compression stockings may be lost to follow-up, go untreated for their CVI, and ultimately, may go on to develop worsening of the disease and ulceration. These patients represent a therapeutic gap in venous disease management. Therefore, there is a need to find alternative therapeutic options that can promote compliance. The manifestations of advanced CVI can significantly affect patient quality of life due to both physical symptoms and the psychosocial burden of disease. In addition, donning compression stockings that are uncomfortable and difficult to use may reduce quality of life. This burden on quality of life correlates with decreased patient compliance with treatment. If a device can deliver compression therapy to the lower extremity and promote quality of life, patients may be more compliant with therapy over time.

**Figure 1:**
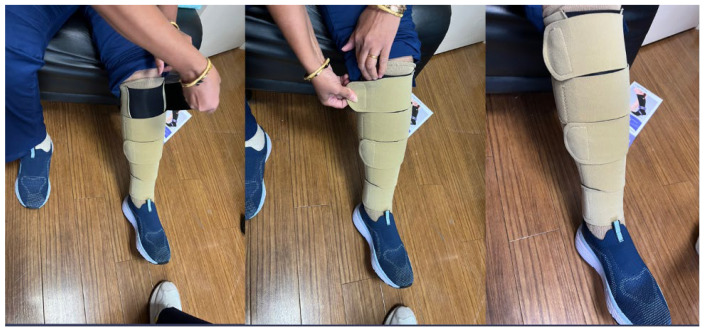
Inelastic compression wrap device.

A novel, inelastic, compression wrap device has been developed as a possible solution to this clinical gap. These wraps promote an easy-to-use, adjustable, therapeutic compression that can be self-applied and can be adjusted to a patient’s comfort level. The wrap consists of series of Velcro straps wrapping circumferentially around the lower extremity, beginning at the ankle and rising to the high calf, just below the knee. The specific compression device used in this study utilizes an ankle to knee compression design. Each compression wrap device is supplied with two sock-liners, which extend from the foot to below the knee. The sock-liners offer compression in the foot, and the compression wrap device offers graded compression from the ankle to below the knee. The compression wrap device, as well as the sock-liners, are washable. The design allows clinicians to prescribe compression at varying therapeutic levels, with the aid of stickers indicating the levels of applied pressure in mmHg (Figure 1). The compression wrap offers the following compression levels, 20–30, 30–40, or 40–50 mmHg of pressure, which can be selected by adjusting how tightly the Velcro straps are fastened. Often, it takes time for patients to gradually adjust to higher pressures of compression. The compression wrap allows clinicians to prescribe varying levels of compression in the same device, eliminating the need for multiple devices and saving patients time and money. Here, we investigate the impact of the novel, inelastic compression wrap device on quality of life (QoL) in patients with CVI who have failed therapy with compression stockings in the past.

## 2. Materials and Methods

We conducted an open-label, single-center, non-blinded, prospective cohort study. Following ethical approval (UM ID 20210807, 8 September 2021) and registration (NCT05051540, 10 September 2021), adults with clinical manifestations, etiology, anatomic distribution, and pathophysiology (CEAP) criteria stages 3–5 CVI (Table 1) who were non-compliant or unsuccessful with compression stockings in the past were eligible to enroll in this study [1].

Adults were recruited to the study by the mechanism of referral in the outpatient clinical setting. Clinicians and researchers who attended wound and dermatology clinics recruited patients based on eligibility as described above. Patients who met the eligibility criteria were informed that they were eligible to take part in a study investigating a novel compression wrap device which would take place over 6 weeks. Patients would be provided with one compression wrap device to utilize for a unilateral study. Patients were informed that the compression wrap device was free, and after completion of the 6-week clinical trial, they could keep and continue to use the compression device free of charge. All patients who were both eligible to participate and interested in the trial were scheduled to meet with a member of the research team at the clinical research office located at the University of Miami Miller School of Medicine for consent and day 1 of the study. Patients completed in-person and phone study visits individually and were not grouped.

**Figure 2:**
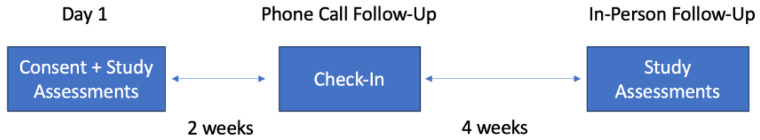
Study outline.

On day 1 of the study, consent was obtained, study assessments were performed, and patients were provided with the novel inelastic compression wrap device (Compreflex™, Sigvaris Inc., Peachtree City, GA, USA). Patients were offered careful instruction regarding how to place the device, adjust the amount of pressure applied, and remove the compression wrap device during the day 1 visit. Patients were instructed to don the wraps in the morning after waking up, wear the wraps consistently all throughout the day, and then remove the wraps at night before bedtime. Two liner socks were included with the compression wrap to act as a barrier between the skin and the compression wrap. Patients were then followed for the next 6 weeks, via a phone call follow-up visit at 2 weeks and an in-person follow-up visit at 6 weeks (Figure 2). A member of the research team called each patient after 2 weeks for the phone call follow-up visit. The intention of the phone call visit was to check in with the patient, answer questions or troubleshoot about the device, remind patients about their scheduled in-person follow-up visit, and assess compliance. The in-person follow-up visit was completed at the same location as day 1.

The primary endpoint of the study was the change in QoL over 6 weeks of therapy with the compression wrap device as measured by the Chronic Venous Disease Quality of Life Questionnaire (CIVIQ-20). Secondary outcomes of the study included measurements of calf circumference at the widest point of the high calf, reported levels of itch, Venous Clinical Severity Scoring (VCSS), and the Short Form Survey-36 (SF-36) questionnaire. Study assessments were performed on day 1 and at week 6. Continuous enrollment took place until thirty patients completed all visits of the study. The study followed an intent-to-treat model. Study participants who completed all study visits, including those who were non-compliers, were included in the analysis. After the study was completed, statistical analysis was performed, including summary statistics, utilizing Excel.

## 3. Results

**Figure 3:**
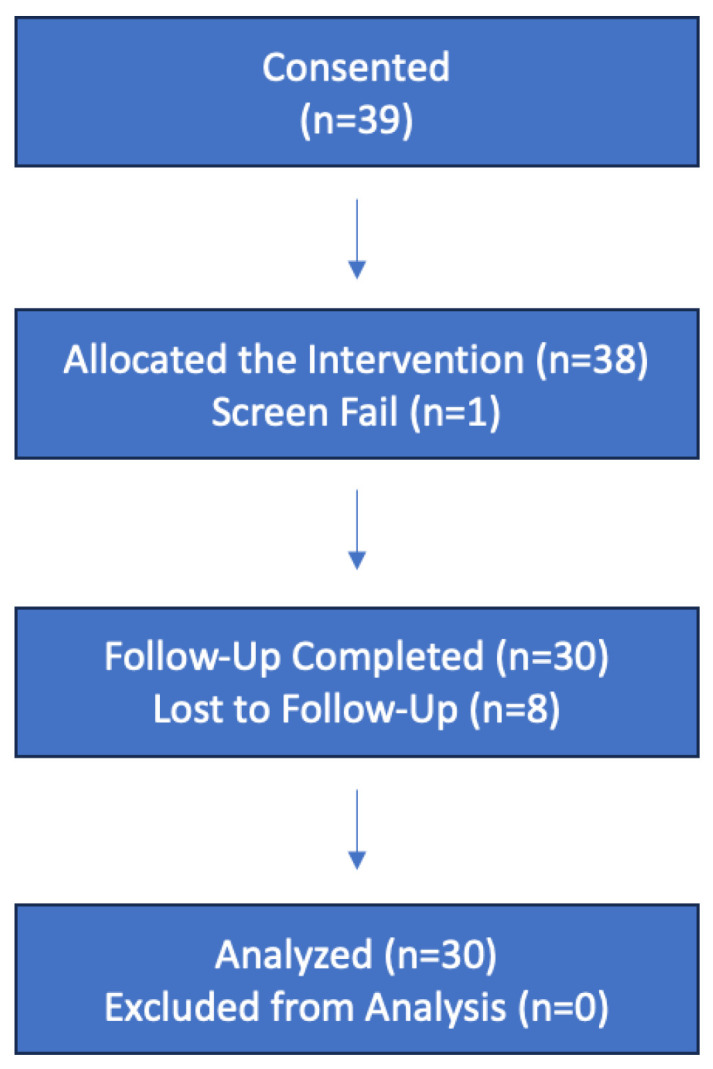
Flow diagram (TREND statement).

From November 2021 to March 2023, 39 patients consented to the study. A total of 30 patients completed both baseline and follow-up visits, 8 patients were lost to follow-up, and 1 patient failed screening (Figure 3). Some of the reasons why eight patients were lost to follow-up include lack of transportation and comorbid conditions as a barrier to compliance in the study. Of the 30 patients who completed the study, 16.7% reported not wearing the wraps consistently (n = 5), while 83.3% reported wearing them consistently in the daytime (n = 25). Of the 30 patients who completed the study, 56.7% (n = 17) were females, and 43.3% (n = 13) were males (Table 2). The ages ranged from 46 to 90, with an average age of 65.2 ± 9.9. The baseline CVI severity mode was C_4a_ on the CEAP criteria scale. The study population featured a higher percentage of female participants than male, and a higher percentage of those older than 65 than those younger than 65. The female sex and advancing age are two risk factors associated with the development of CVI [11,12,13]. This is reflected in the study population and can be seen in the target population of individuals with CEAP stages C_3_–C_5_.

The primary outcome of the study was the CIVIQ-20 questionnaire (Table 3). At the six-week follow-up, CIVIQ-20 scores improved on average 12.123 ± 21.056 points on a 100-point scale (*p* = 0.0019).

Calf circumference decreased on average 1.3 cm ± 2.21 cm over the six weeks (*p* = 0.0009). Reported levels of average itch on a ten-point scale decreased on average by 1.9 ± 2.63 points (*p* = 0.0008) and reported levels of the worst itch on a ten-point scale decreased on average by 2.73 ± 3.63 points (*p* = 0.0001). The VCSS scores, which are on a 30-point scale, ranging from least severe to most severe clinical venous disease, decreased on average by 1.276 ± 2.297 points (*p* = 0.0029). The SF-36 questionnaire is organized into nine domains, including pain, physical functioning, social functioning, health change, general health, role limitations due to physical health, energy/fatigue, role limitations due to emotional problems, and emotional well-being. The total points possible in each category on the SF-36 is 100, and a higher score indicates a higher functioning in that category. The only category with a statistically significant change over the six weeks on-study was in pain. Pain scores improved on average by 7.76 ± 21.55 points (*p* = 0.0313). Several other SF-36 domains demonstrated improvement, however, they did not meet statistical significance. Two domains, role limitations due to emotional problems and emotional well-being, demonstrated slight worsening, however these outcomes were statistically insignificant. Patient verbal feedback was also collected at the conclusion of the study. There were no adverse events reported during this study. Patients were overall very satisfied with the look, function, and feel of the device.

## 4. Discussion

Chronic venous insufficiency is treated with compression, traditionally with the utilization of compression stockings. Compression therapy is a longitudinal treatment, due to the chronic nature of venous disease, that requires significant patient participation. One study found that the single most important belief that distinguished patients who wore compression stockings versus those who did not is the belief that compression therapy is worthwhile [14]. Patients must believe that compression will benefit them over time in order to be willing to wear them day after day over months and even years. The same study also found that the single most important belief that identified patients who were not to be compliant with compression stockings was the belief that wearing stockings is uncomfortable. Finding an alternative therapy that delivers compression in a comfortable way may increase overall patient participation in their own health and CVI treatment.

Here, we demonstrated that over the course of six weeks of use of the novel, inelastic compression device, patients displayed an improvement in quality of life, as demonstrated by the CIVIQ-20 questionnaire. Use of the compression device was also correlated with an improvement in several other clinical metrics, such as levels of itch, the VCSS score, calf circumference, and pain. This constellation of improvement in both the QoL and clinical measures suggests that the effect of the intervention is real and makes this device an important addition to the treatment toolbox for CVI.

We used the CIVIQ-20 questionnaire to discern QoL improvement. The CIVIQ-20 Global Index Score (GIS) ranges from 0 to 100, with higher scores indicating a higher quality of life. In the literature, the mean CIVIQ-20 score for people with CVI stage C_3_ is 59.70, and for C_4_ it is 57.95 [15]. The mean baseline CIVIQ-20 score in the population we assessed, across CEAP stages C_3_–C_5_, was 59.04. This is similar to baseline scores in the literature, indicating that the QoL burden of CVI on our patients is representative. Across the literature, CVI interventions have yielded a wide range of impact on CIVIQ-20 scores. Examples of mean CIVIQ-20 score differences seen with CVI interventions are +39.36 (exercise after 8 weeks [16]), +10.6 (cryostripping in the setting of CVD after 6 months [17]), and +43.9 (subcutaneous lidocaine injection after 1 month and 6 months [18]). One study that evaluated iliac venous stenting for CVI found no statistically significant difference in mean CIVIQ-20 scores between treated and control cohorts at about one year following procedural intervention [19]. Over six weeks of intervention with the inelastic compression wrap device, we found a statistically significant mean change of 12.12 points on the CIVIQ-20 score from baseline. Considering that this is an affordable, non-invasive intervention, this represents a meaningful improvement. The device is simple to use, comfortable, and can be utilized by patients from the convenience of their own homes.

The SF-36 questionnaire also utilizes a series of questions to evaluate quality of life, however, it is nonspecific to chronic venous insufficiency (CVI). There are nine domains, and these assess both physical and emotional health changes. Across the literature, SF-36 questionnaires have been utilized as a metric to evaluate success of CVI interventions through representing patient QoL over time [16,20,21]. However, intervention, patient numbers, follow-up windows, baseline inclusion criteria, and quality of evidence vary greatly. The only domain with a statistically significant change in our study was pain, with an improvement across the six weeks of study. Five of the remaining seven domains demonstrated an improvement, and two of the remaining seven domains demonstrated a slight worsening, but none were statistically significant. Possible reasons include the short time spent on the study and the variance in baseline comorbidities. The SF-36 domains may have been too broad, such as the category of “general health”, to significantly change in only six weeks when patients suffer from a variety of comorbidities. Notably, the five subjects who did not wear the wraps consistently were included in an intention-to-treat model and could have modified our results.

A significant barrier to patient compliance for compression stockings is the common belief that they are uncomfortable and not worthwhile. Quality of life is a key metric in the success of a compression device over time. Compliance rates as low as 11% and as high as 97.2% have been documented in the literature [22,23]. However, compliance rates across several studies averaged about 62% [23]. Here, we report a compliance rate of 83.3% with the novel compression device. Although over a short time period, we note this rate is high compared to the wide range of compliance rates in the literature. Uniquely, this is especially impressive because each patient included in this study endorsed non-compliance with compression stocking therapy prior to starting treatment with the compression wrap. Therefore, this cohort represents a highly treatment-resistant group.

Itch is a symptom that has been positively correlated with increasing severity of CEAP stages [24]. In addition, one study also demonstrated that leg or feet itch in chronic venous disease was associated with a poorer level of health-related quality of life, increased levels of leg pain, and a higher level of comorbid conditions [24]. In this study, itch was quantified as a patient-reported score out of 10, with 10 being the worst possible itch, and 0 being the lack of any itch. Patients were asked to report the average level of itch (out of a score of 10) and the worst level of itch (the highest score out of a score of 10 that a patient ever experiences on a given day) at day 1 and week 6. Notably, both the average level of itch and worst level of itch decreased with statistical significance over the course of the study.

The VCSS tool has been correlated with CEAP stages, the modified CIVIQ questionnaire, and venous ultrasound findings, and it offers an additional metric to quantify severity of CVI via physical exam and patient interview [25]. Here, use of a non-invasive, low-risk compression device over a six-week period resulted in a mean reduction in VCSS scores of 1.3 ± 2.3. Achieving VCSS score reduction with a non-invasive compression device highlights the need to choose adequate compression interventions.

A limitation to our study is the short window for improvement as some domains of the SF-36, such as general health, may require a longer time period for evaluation of the effect. Another limitation is that our study was monocentric. Additional limitations include the lack of a control arm and control for comorbid conditions; however, each subject was their own control, and the short duration of the study may help to limit the effect of other interventions. Additionally, the constellation of positive effects noted (in both clinical and QoL assessments) suggest a real effect due to the intervention. Finally, a number of patients did not wear the wraps consistently. Reasons that patients reportedly did not comply with therapy included patient-dependent factors, irritation to the skin, limited mobility, lack of support in the home, baseline lower extremity pain from neuropathy, and dislike of the appearance of the wrap. However, the majority of patients did well with the compression devices and wore the compression wrap as directed. Notably, a compliance rate of 83% is very high for compression devices in this population.

Compression stockings remain the mainstay of treatment for advanced cutaneous manifestations of CVI. However, we demonstrated that the novel inelastic compression device offers an alternative and may improve QoL, pain, itch, and compliance in a safe manner in people with CVI who could not tolerate compression stockings.

## Figures and Tables

**Table 1:** CEAP criteria [1].

**C Class [1]**	**Description**
C_0_	No visible or palpable signs of venous disease
C_1_	Telangiectasias or reticular veins
C_2_	Varicose veins
C_2r_	Recurrent varicose veins
C_3_	Edema
C_4_	Changes in skin and subcutaneous tissue secondary to CVD
C_4a_	Pigmentation or eczema
C_4b_	Lipodermatosclerosis or atrophie blanche
C_4c_	Corona phlebactatica
C_5_	Healed venous ulcer
C_6_	Active venous ulcer
C_6r_	Recurrent active venous ulcer

**Table 2:** Study demographics.

Demographics	Number of Participants
**Age**	
30–49	1
50–64	12
65+	17
**Gender**	
Male	13
Female	17
**CEAP Stages**	**At Baseline**
C_3_	2
C_4a_	11
C_4b_	4
C_5_	13

**Table 3:** Study outcomes.

Study Metric	Average Change from Baseline at Conclusion of Study (Mean Change ± Standard Deviation)	*p*-Value (n)
CIVIQ-20 Questionnaire (100 point scale)	12.123 ± 21.06	0.0019
Calf Circumference in Centimeters	−1.3 ± 2.21	0.0009
Reported Levels of Average Itch on 10 Point Scale	−1.9 ± 2.63	0.0008
Reported Levels of Worst Itch on 10 Point Scale	−2.73 ± 3.63	0.0001
VCCS Score (30 Point Scale)	−1.276 ± 2.30	0.0029
SF-36 Questionnaire: Pain Domain (100 Point Scale)	7.76 ± 21.55	0.0313
SF-36 Questionnaire: Physical Functioning Domain		0.0592
SF-36 Questionnaire: Social Functioning Domain		0.0829
SF-36 Questionnaire: Health Change Domain		0.0936
SF-36 Questionnaire: General Health Domain		0.1348
SF-36 Questionnaire: Role Limitations Due to Physical Health Domain		0.1968
SF-36 Questionnaire: Energy/Fatigue Domain		0.4348
SF-36 Questionnaire: Role Limitations Due to Emotional Problems Domain		0.1414
SF-36 Questionnaire: Emotional Well Being Domain		0.3447

## Data Availability

Study data are owned by the University of Miami and requests may be made by contacting hlevtov@med.miami.edu.
